# Fragmentation in mitochondrial genomes in relation to elevated sequence divergence and extreme rearrangements

**DOI:** 10.1186/s12915-021-01218-7

**Published:** 2022-01-07

**Authors:** Shiqian Feng, Andrea Pozzi, Vaclav Stejskal, George Opit, Qianqian Yang, Renfu Shao, Damian K. Dowling, Zhihong Li

**Affiliations:** 1 Department of Plant Biosecurity, College of Plant Protection, China Agricultural University, Beijing, 100193 China; 2 School of Biological Sciences, Monash University, Clayton, VIC 3800 Australia; 3 Crop Research Institute, Drnovská 507, 161 06 Prague, Czech Republic; 4 Faculty of Agrobiology, Food and Natural Resources, Czech University of Life Sciences, Kamycka 129, 165 00 Prague, Czech Republic; 5 Department of Entomology and Plant Pathology, Oklahoma State University, Oklahoma, 74078 USA; 6 Zhejiang Provincial Key Laboratory of Biometrology and Inspection & Quarantine, College of Life Sciences, China Jiliang University, Hangzhou, 310018 China; 7 GeneCology Research Centre, Centre for Animal Health Innovation, School of Science and Engineering, University of the Sunshine Coast, Maroochydore DC, Queensland 4556 Australia

**Keywords:** Mitochondrial genome, Booklice, Fragmentation, Rearrangement, Recombination, Evolution

## Abstract

**Background:**

A single circular mitochondrial (mt) genome is a common feature across most metazoans. The mt-genome includes protein-coding genes involved in oxidative phosphorylation, as well as RNAs necessary for translation of mt-RNAs, whose order and number are highly conserved across animal clades, with few known exceptions of alternative mt-gene order or mt-genome architectures. One such exception consists of the fragmented mitochondrial genome, a type of genome architecture where mt-genes are split across two or more mt-chromosomes. However, the origins of mt-genome fragmentation and its effects on mt-genome evolution are unknown. Here, we investigate these origin and potential mechanisms underlying mt-genome fragmentation, focusing on a genus of booklice, *Liposcelis*, which exhibits elevated sequence divergence, frequent rearrangement of mt-gene order, and fragmentation of the mt genome, and compare them to other Metazoan clades.

**Results:**

We found this genus *Liposcelis* exhibits very low conservation of mt-gene order across species, relative to other metazoans. Levels of gene order rearrangement were, however, unrelated to whether or not mt-genomes were fragmented or intact, suggesting mitochondrial genome fragmentation is not affecting mt-gene order directly. We further investigated possible mechanisms underpinning these patterns and revealed very high conservation of non-coding sequences at the edges of multiple recombination regions across populations of one particular *Liposcelis* species, supportive of a hypothesis that mt-fragmentation arises from recombination errors between mt-genome copies. We propose these errors may arise as a consequence of a heightened mutation rate in clades exhibiting mt-fragmentation. Consistent with this, we observed a striking pattern across three Metazoan phyla (Arthropoda, Nematoda, Cnidaria) characterised by members exhibiting high levels of mt-gene order rearrangement and cases of mt-fragmentation, whereby the mt-genomes of species more closely related to species with fragmented mt-genomes diverge more rapidly despite experiencing strong purifying selection.

**Conclusions:**

We showed that contrary to expectations, mt-genome fragmentation is not correlated with the increase in mt-genome rearrangements. Furthermore, we present evidence that fragmentation of the mt-genome may be part of a general relaxation of a natural selection on the mt-genome, thus providing new insights into the origins of mt-genome fragmentation and evolution.

**Supplementary Information:**

The online version contains supplementary material available at 10.1186/s12915-021-01218-7.

## Background

Mitochondria are present in virtually all multicellular eukaryotes, and their function is to provide energy for the cell [1]. Energy conversion is regulated by a series of five enzyme complexes, which comprise oxidative phosphorylation (OXPHOS), whose subunits consist of a combination of proteins encoded by the nuclear and mitochondrial genomes [2, 3]. The mitochondrial genome (mt-genome) usually exists as a small circular chromosome in bilaterians (~ 16 kb length) that harbours only a few protein-coding genes necessary for OXPHOS and the RNAs necessary for the translation of mt-mRNAs [1, 2]. The number of these genes found in the mtDNA sequence, and their order, are highly conserved across animals, thus making any occurrence of gene rearrangement among these genes useful for phylogenetic analysis [4]. Indeed, rare gene rearrangements often mark differences between clades, making the study of mt-gene order a valuable tool for studying deep phylogeny [4–6]. However, curiously, not all clades and species exhibit highly conserved mt-gene order. In fact, the huge increase in the number of sequenced genomes across metazoan taxa over the past decade has brought to light some species exhibiting atypical mt-genomes, where neither mt-gene order nor the mt-genome architecture is conserved.

These atypical mt-genomes exhibit changes of mt-gene order across closely related congeneric species, occasionally exhibiting stark changes in architecture from a single circular chromosome to fragmentation into multiple circular mt-chromosomes or mt-genome linearization. These changes in mt-genome architecture are even rarer than gene rearrangements among the metazoan phylogenies. However, there are two exceptions, in which gene order rearrangements and fragmentation are relatively common: in lice (insects from the insect order Psocodea) and cnidarians (including jellyfish, corals, anemones and other stingers) [7]. In 2019, the human body louse *Pediculus humanus* was shown to possess a fragmented mt-genome with 20 minichromosomes [8]. Indeed, approximately 30 species of parasitic lice and booklice (two clades in Psocodea) have been found to possess fragmented mt-genomes with 2–20 minichromosomes [9, 10]. Cnidarians represent the other exception, with species in this phylum exhibiting a wide range of atypical mt-genome architectures, spanning from the normal circular genome to fragmented linear genomes [7, 11]. A unique example among species in this phylum with atypical mt-genome architecture is a group of parasitic cnidarians (Myxozoa) with circular fragmented mt-genomes [11]. The Myxozoa mt-genome has fewer genes than other cnidarian mt-genomes, and these genes are spread across different mt-chromosomes, resembling fragmentation from an ancestral circular mt-genome to multiple smaller fragments. However, the occurrence of atypical mt-genome architectures, such as fragmented circular mt-genome, is not limited to Insecta and Cnidaria, with recent studies demonstrating similar patterns occurring in a few other clades, such as Porifera [12] and Nematoda [13–16].

Currently, it remains challenging to explain how these extreme modifications to mt-genome architecture have evolved in metazoans, and why they are limited to just a handful of species occurring across disparate phyla, including both nonbilaterians and bilaterians. Some recent insights have, however, been achieved by researchers studying an abundance of sequenced linear mt-genomes in Cnidaria, who have identified specific homologous genes shared across all species with linear mt-genomes. This suggests linearization of mt-genomes was caused by the incorporation of a linear plasmid with the ability to linearize a circular mt-chromosome, at least in Cnidaria [7, 17]. Unfortunately, similar data that might provide insights into the evolution of circular fragmented mt-genomes in other clades are scarce, with researchers thus far only making tentative speculation as to the drivers of mt-genome fragmentation [16, 18]. These speculations have been mostly done by the pioneers who first discovered these mt-chromosomes in multiple species [16, 18]. One study described the occurrence of fragmented mt-genomes (consisting of two circles) in the genus *Globodera* (Nematoda), with the authors proposing that fragmentation into two mini-circles would have occurred by a whole genome duplication, followed by purifying selection leading to the extinction of specific genes on each mini-circle, ultimately resulting in multiple mt-chromosomes with different genes [16]. On the contrary, the researchers discovering the 20 mt-minicircles in *Pediculus humanus* (Arthropoda) proposed a mechanism in which the original mt-genome is slowly replaced by smaller mt-chromosomes that replicate faster [18]. Notwithstanding, both the underpinning mechanisms and evolutionary processes that would mediate mt-genome fragmentation in the first instance remain completely elusive.

Therefore, we examined these putative mechanisms and evolutionary processes by performing a comparative analysis across three clades of metazoans with fragmented mt-genomes, and proposed a hypothesis based on our observations. In the comparative analysis, we investigated three different features of mitochondrial biology that might relate to mt-genome fragmentation. These features were the degree of mt-gene rearrangement, sequence divergence and the signature of selection acting on these genes. Our comparative analysis combined a mix of new data collected from the booklice, *Liposcelis*, and leverages existing datasets across several phyla. The incorporated data from *Liposcelis* enabled us to examine processes of mt-fragmentation at high evolutionary resolution, homing in on intraspecific differences in mt-genome architecture across the *Liposcelis* genus, and indeed probing intraspecific differences in patterns of mt-fragmentation across populations of one particular species, *Liposcelis bostrychophila*. By combining multiple datasets and measurements on mt-genome fragmentation, our results give rise to a new hypothesis and provide unique insights into the drivers of mitochondrial genome evolution.

## Results

### The booklice mt-genome has highly variable gene order and genome architecture

**Fig. 1:**
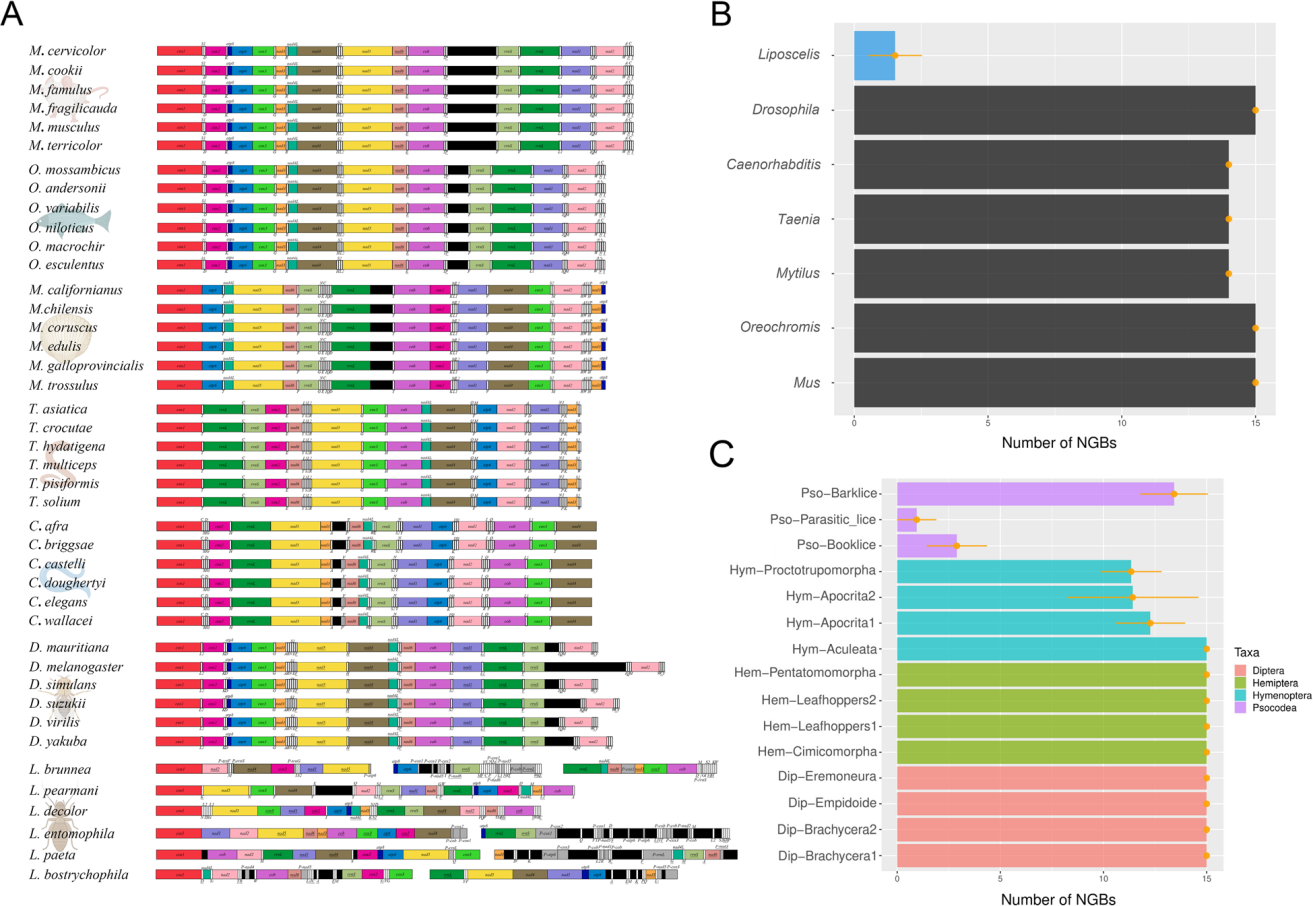
Extraordinary mt-gene rearrangements were found in booklice compared to different bilaterian clades. **A** Linearized mt-genome arrangements across six “well-known” genera. They are *Mus* and *Oreochromis* representing Chordata, *Mytilus* from Mollusca, *Taenia* from Rotifera, *Caenorhabditis* from Nematoda, *Drosophila* from Arthropoda. We also show the divergent mt-genome organisation from our focal genus *Liposcelis*. **B** Number of gene boundaries (NGBs) of the six representative genera of bilaterians which incorporates together all sequences from the six representative genera. This panel highlights the observation that booklice (*Liposcelis*) have many more mt-gene rearrangements (i.e. low level of NGBs). **C** NGBs of four orders in Insecta. This panel compares the level of mt-gene rearrangements across groups of taxa of similar size and age (~ 10 species and ~200 Mya old, see Additional file 2: Table S1). Each of the four clades includes species from only one Insecta order (indicated by different colours)

First, we conducted a comparative analysis examining levels of mt-gene rearrangement across seven genera spanning five different phyla, finding that rearrangements in the booklouse genus *Liposcelis* are much less conserved than six other well-studied genera (Fig. 1A). The clades analysed here have been the focus of many studies, thus providing us with multiple high-quality genomic sequences and reliable gene order data to compare to data from *Liposcelis*. Despite being part of different phyla, six of the genera across the five phyla compared exhibited highly consistent patterns, sharing the same mt-gene order within each genus, and exhibiting few mt-gene rearrangements across phyla. However, the genus *Liposcelis* was an exception, exhibiting very low conservation in mt-gene order. To better understand the magnitude of mt-gene order conservation, we quantified how often mt-genes changed positions in our dataset by calculating the number of shared gene boundaries (NGBs) across species (the rationale and workflow used for quantifying NGBs are presented in Additional file 1: Fig. S1) within representative clades. We compared the NGBs of booklice to those of the six representative genera (Fig. 1B; Additional file 2: Table S1), showing that the level of mt-gene order conservation in the genus *Liposcelis* (NGBs: 1.53±1.02) is about nine-fold lower than that of the well-studied genera (NGBs: 14.00±0.00 or 15.00±0.00). While the notion of different metazoan clades exhibiting differences in genome stability is not new [19], the magnitude of mt-gene instability we found in *Liposcelis* is unique.

Next, we investigated phylogenetic signals of mt-gene order instability across the taxa close to *Liposcelis*. We restricted the data to species belonging to the class Insecta. We compared the mt-gene order conservation across three well-studied clades within Insecta (Diptera, Hymenoptera, and Hemiptera), as well as lice clades in Psocodea. The clades were chosen with the aim of finding groups of species with a phylogenetic relationship similar in diversity and age to the species of the Psocodea clade. The 15 clades chosen have similar age (~200Mya) and include a similar number of species (~ 10), providing a reasonable comparison to the *Liposcelis* clade (~ 220 Mya and 11 species). The clades names, ages and species included can be found in Additional file 2: Table S1. These clades were then compared to species within *Liposcelis* (Fig. 1C; Additional file 2: Table S1). Through this analysis, we discovered that *Liposcelis* and Phthiraptera had about eightfold fewer NGBs than other clades, and that species more closely related to *Liposcelis* and Phthiraptera*,* such as barklice (NGB = 12.41±1.84)*,* exhibited lower NGBs than more distant clades and the ones in Hemiptera (NGB = 15.00±0.00) or Diptera (NGB = 15.00±0.00). Although species within Hymenoptera exhibited less conserved mt-gene order compared to those within Hemiptera and Diptera, NGBs were still high relative to those within *Liposcelis* (NGB = 11.34±1.47 to 15.00±0.00). The clades *Liposcelis* and Phthiraptera exhibited very fragmented mt-genomes (up to 20 fragments) and the lowest number of NGBs. Based on a hypothesis that mt-genome fragmentation could be a potential mechanism for explaining extraordinary instances of mt-gene rearrangement in metazoans [20], low NGBs are subsequently expected for species with fragmented mt-genomes. However, our analysis highlights that some species within the *Liposcelis* clade have very low NGBs while having a perfectly intact circular mt-genome, such as the newly sequenced *L. pearmani.* Our observations, therefore, do not support this hypothesis and suggest that mt-genome fragmentation may not be the primary driver of the low levels of mt-gene order stability observed throughout this genus.

### Fragmented mt-genomes: the cause or consequence of gene order rearrangements?

**Fig. 2:**
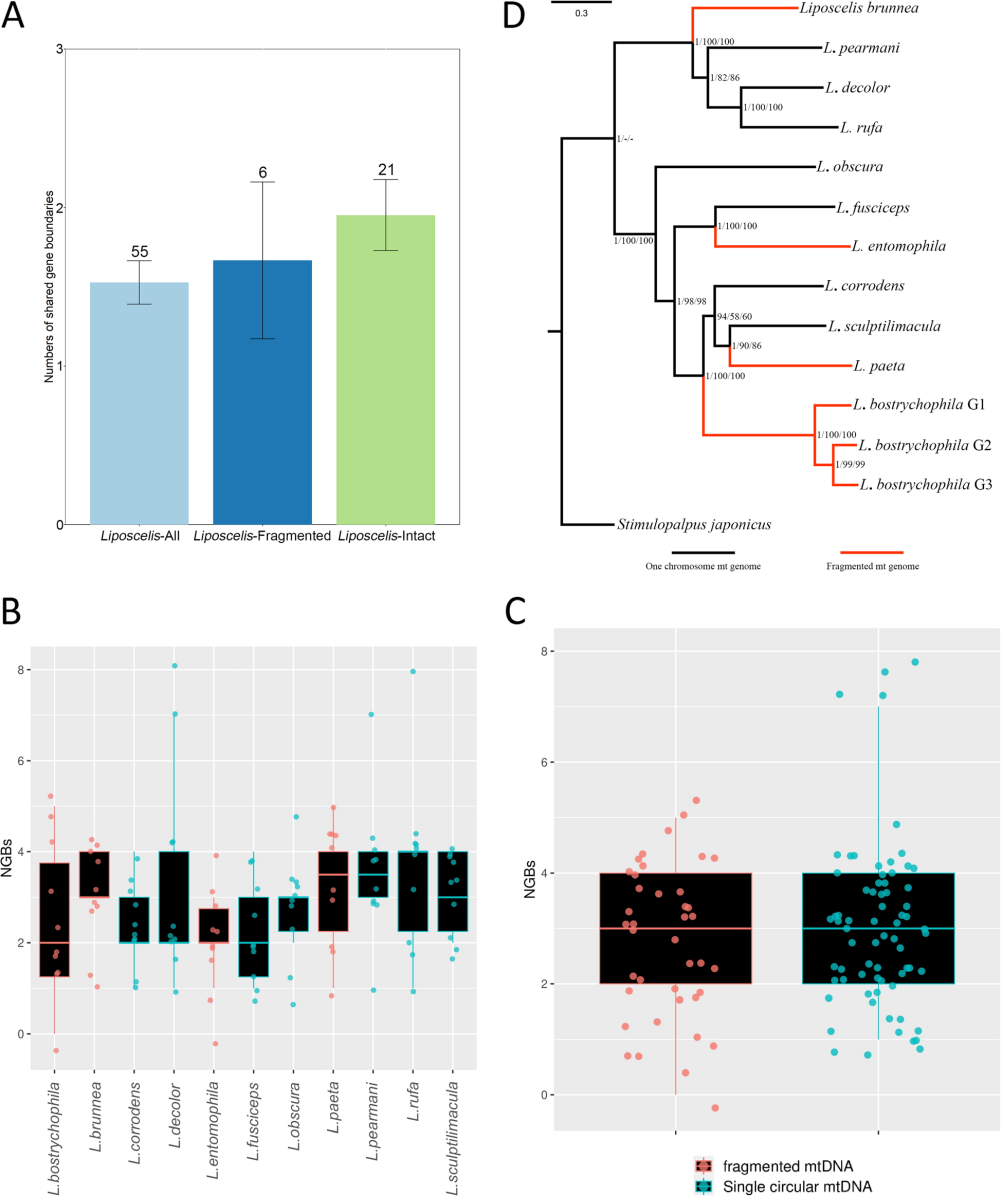
Mt-genome rearrangements and fragmentation in booklice. **A** Comparison of number of gene boundaries (NGBs) between booklice with fragmented or non-fragmented mt-genomes. No significant difference was detected. The number above each error bar is sampling size. **B** A boxplot comparing the mt-genome rearrangements (NGBs count) across the species within the *Liposcelis* genus through pairwise comparisons. Each datapoint represents a comparison between the species considered and one of the other *Liposcelis* species. **C** The figure shows a comparison of mt-genome recombination (using NGBs as a proxy) between all species with fragmented and unfragmented mt-genomes. The datapoints are the same as the previous figure but they have been pooled together and no significant difference was detected. **D** Phylogenetic tree of the genus *Liposcelis* based on PCG123 dataset. The values in each node are posterior probability from MrBayes, bootstrap values based on IQtree and PhyML. Four branches with fragmented mt-genomes are labelled in red. All other trees based on three datasets (PCG123rRNA, PCG12, PCG12rRNA) are reported in Additional file 1: Fig. S2, and confirm the topology shown here

We further investigated the role of mt-genome fragmentation on the conservation of mt-gene order by performing comparative analyses between two groups of *Liposcelis* species with and without mt-genome fragmentation. The analysis revealed similar levels of mt-gene stability between the two groups, with no significant difference in the NGBs between species with intact and those with fragmented mt-genomes (Mann-Whitney *U* test, *p* value = 0.69, NGBs_fragmented_ = 1.67±0.49, NGBs_intact_ = 1.95±0.22, Fig. 2A). Similarly, we computed the number of NGBs of each species using pairwise comparison between all the *Liposcelis* species, finding once again that there is no significant difference (Mann-Whitney *U* test, *p* value = 0.28) between species with fragmented and intact mt-genomes. The lack of differences in mt-gene recombination (using NGBs as a proxy) between fragmented and unfragmented species is evident both in single species (Fig. 2B) and group comparisons (Fig. 2C). These analyses indicate that patterns of mt-gene rearrangement do not differ between fragmented and unfragmented species, therefore suggesting that mt-genome fragmentation is not the likely driver of the high levels of mt-gene order variation observed among some Metazoan clades.

To better understand the patterns of mt-genome fragmentation in the *Liposcelis* genus, we computed a phylogenetic tree based on the mtDNA of all booklice. This analysis revealed a polyphyletic origin of the fragmented mt-genomes within the genus (Fig. 2D; Additional file 1: Fig. S2). We found that each booklouse species with fragmented mt-genome is more closely related to other species with single circular mt-genomes than to other species with fragmented genomes, indicating that mt-genome fragmentation occurred on four independent occasions within the genus. However, we believe that so many independent episodes of the evolution of fragmentation are unlikely, and we argue that the presence of mt-genome fragmentation scattered across the *Liposcelis* phylogenetic tree suggests instead that mt-genome fragmentation might be a rare consequence of another process that is widespread throughout the whole clade. Thus, we proposed a hypothesis to explain our phenomenon. Specifically, we hypothesise that the process leading to mt-fragmentation is the presence of widespread recombination in *Liposcelis* mt-genomes; recombination could in theory account for both the high level of gene order rearrangement across the mt-genomes of all species within the genus, and the sporadic mt-genome fragmentation in some species.

### The potential role of recombination in intraspecific mt-genome structural variation

**Fig. 3:**
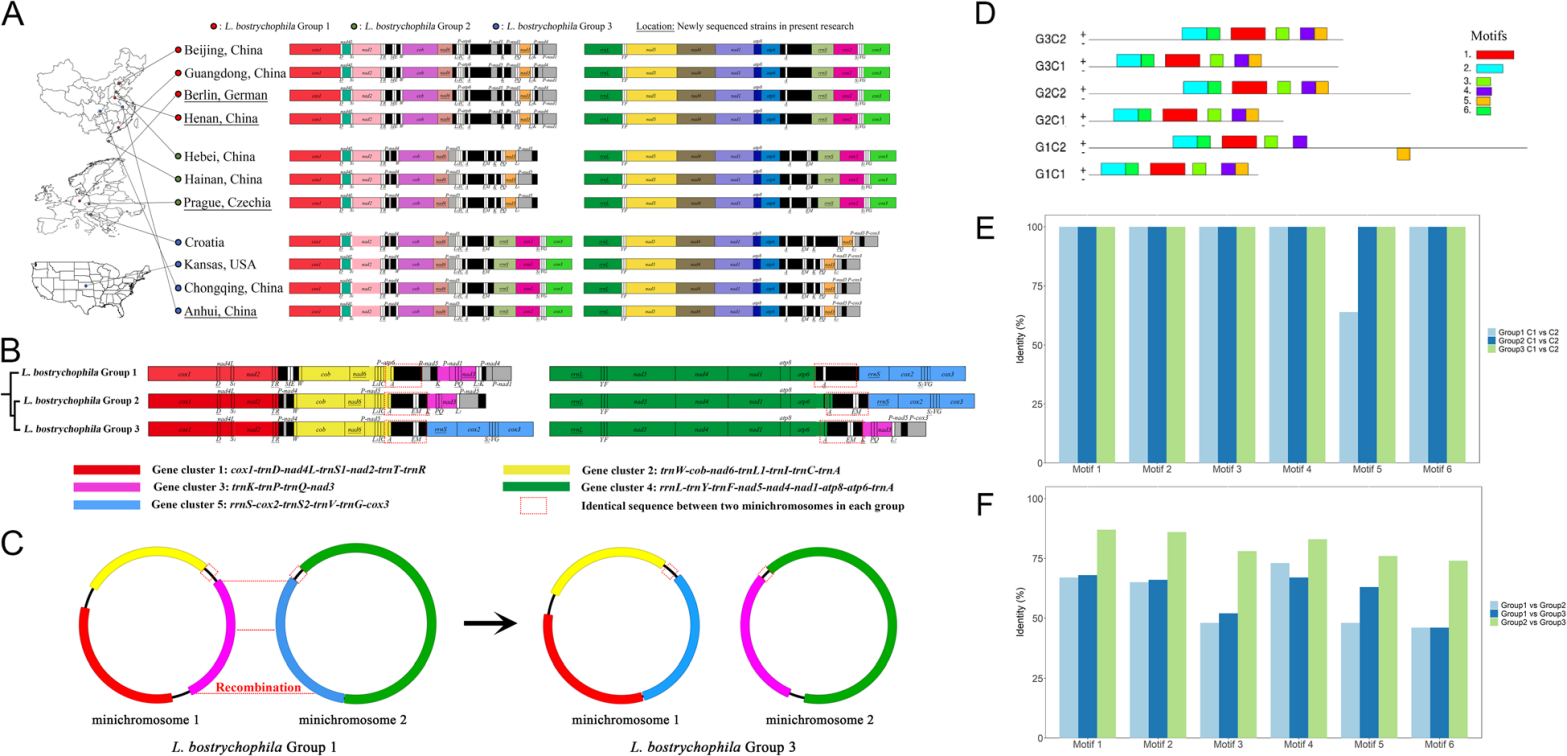
Mt-genome sampling, inter-molecule recombination and non-coding region of *Liposcelis bostrychophila.* **A** Sampling locations of the three *L. bostrychophila* groups. The three groups of *L. bostrychophila* dispersed randomly across three continents. **B** The mitochondrial genome arrangements of three *L. bostrychophila* groups. Gene clusters are highlighted using different colours, showing that groups 1 and 2 have similar gene cluster arrangement, different from group 3. **C** Homologous recombination happened between two mt-chromosomes of *L. bostrychophila*. Four to nine mt-genes constitute five conserved gene clusters in all three groups of *L. bostrychophila*. Based on the phylogeny among the three groups, group 3 is derived from group 1. The group 3 arrangement may be created via inter-molecule recombination between the two mt-chromosomes of group 1. **D** The motifs identified in non-coding regions. The mt-chromosomes were named by their location and group, for example, G1C1 means group 1(G1) mt-chromosome 1(C1). Six motifs with the same arrangement were found in both mt-chromosomes of the three groups while only the Motif5 in G1C2 (mt-chromosome 2 of group 1) translocated to the other strand. The strands are indicated by using the signs plus and minus. **E** A comparative analysis of the identities between motifs in the minichromosomes C1 and C2. All motifs had identical sequences between two mt-chromosomes in each group with the exception of Motif5 (group 1). **F** A comparative analysis of the identities between motifs in groups 1, 2 and 3. The motif similarities between group 2 and group 3 are higher than their comparison to group 1. The result here is according to the phylogeny-based relationship

To investigate whether recombination might explain the phenomena observed, we focused on a single species of this clade, *L. bostrychophila*, exhibiting intraspecific mt-gene rearrangements across populations [21]. Although we cannot exclude the possibility of cryptic species within the populations of *L. bostrychophila*, we treated these populations as belonging to one species, as this is consistent with the available molecular phylogenetic and morphological evidence [22]. We investigated intraspecific patterns of the mt-gene rearrangements in this species by combining newly sequenced mt-genomes with sequences already available from multiple populations of *L. bostrychophila*. We increased the existing pool of sequenced populations for this species by sequencing samples from worldwide locations previously not sampled and verified the presence of three known distinct mt-gene arrangements (groups 1, 2, and 3) across these populations (Fig. 3A). The three groups are defined by specific and distinct gene orders across the mt-genomes, present across populations of *L. bostrychophila*. In the new sampling locations, we found that only these three known groups are present. In each of the populations sampled, we found that all mt-genomes were fragmented into two minichromosomes. And within each of the three groups of mt-gene arrangement, the intra-group sequence conservation was almost 100%, including within non-coding regions. The presence of recombination without sequence divergence is quite rare in animals, however, is more common in plant mitochondrial genomes, especially when mt-genomes have conserved sequence repeats [23], suggesting that the *Liposcelis* mt-genome might have some sequence that makes the genome more prone to recombination.

We further explored signatures of structural variation in the mt-genome across the three *L. bostrychophila* groups, to investigate whether recombination may underpin the high levels of mt-gene order rearrangement specifically within this species, and generally observed across the genus. We confirmed the presence of five conserved gene clusters across the three *L. bostrychophila* groups, with four to nine mt-genes in each cluster [21]. The two mt-chromosomes of group 1 and group 2 have the same gene cluster distribution (Fig. 3B), whereby gene clusters 1, 2, and 3 are located on mt-chromosome 1, while gene clusters 4 and 5 are located on mt-chromosome 2. In contrast, in group 3, gene clusters 1, 2, and 5 are located on mt-chromosome 1, while gene clusters 3 and 4 lie on mt-chromosome 2. Based on the phylogenetic analyses shown before, group 1 represents the ancestral lineage of group 2 and 3. These patterns of gene cluster synteny can be explained by the exchange of gene cluster 3 and gene cluster 5 through inter-molecule recombination in group 1, leading to the mt-gene order observed in group 3 (Fig. 3C). Therefore, recombination could be a perfect candidate in the explanation of mt-genome structural variation.

### Some non-coding regions of the mt-genome show higher conservation than the protein-coding regions

We also noticed that the non-coding regions near the gene clusters 3 and 5 (highlighted in red dash boxes in Fig. 3B, C) are particularly conserved in each group, suggesting that these regions might be important in understanding patterns of mt-gene recombination and fragmentation. To further investigate a putative role of these non-coding regions in mediating recombination in the mt-genome, we performed comparative analyses across the three *L. bostrychophila* groups, with the aim of identifying conserved motifs within these regions. By comparing the non-coding regions of the two minichromosomes across three groups, we identified six conserved motifs that are shared by each group in the same order (Fig. 3D). These motifs are short mtDNA regions of less than 100 nt, with highly conserved sequence (~ 100%) and unknown function. While the mt-chromosomes of each group all harbour the same sequence of DNA motifs, there is one exception, the second mt-chromosome in the first group (G1C2). In this instance, one of the conserved motifs in the non-coding region (which we called Motif 5) has relocated further along the non-coding region of mt-chromosome 2 (C2). Furthermore, G1C2 Motif 5 has a sequence identity of only 64% when comparing G1C1 and G1C2, thus being the least conserved of the motifs (Fig. 3E). As the DNA motifs with conserved positions have 100% sequence identity across chromosomes, it is possible that the position within the non-coding region might affect the conservation of the sequence. Although the sequence conservation of the DNA motifs is constant across mt-chromosomes in the same groups, it varies when comparing motifs from different groups, with groups 2 and 3 sharing higher conservation in motif sequence identity than comparisons of these groups to group 1 (Fig. 3F). Notwithstanding, the high conservation of these supposedly non-functional DNA motifs is puzzling, especially considering that this kind of motifs are not unique to this species. Indeed, a similar pattern has been observed before in a related clade (Columbicola, family Philopteridae) where the species have very fragmented mt-genomes but still share conserved putatively non-functional motifs [24].

To further probe the putative function of these conserved DNA motifs, we scanned the blastn database for similar sequences and analysed the motifs for potentially meaningful open reading frames (ORFs). The few hits obtained from the blast search were with proteins unrelated to the mitochondria, with e-values of around 0.002, which although formally considered to be meaningful [25], we believe are unreliable in this context given the result is so unusual (Additional file 2: Table S2). Likewise, the ORF finder identified at least one ORF with meaningful matches in the blast database for the first 4 motifs, with *e*-values between 0.01 and 0.0003 for proteins that we would not expect in the mt-genome (ORF finder results in Additional file 2: Table S3). As the biological information of the hits does not match the context of these analyses, we believe that none of these hits is meaningful, thus suggesting that these DNA motifs might be conserved for reasons other than translation into functional proteins.

### Enhanced sequence divergence indicates structural variation in mt genomes

**Fig. 4:**
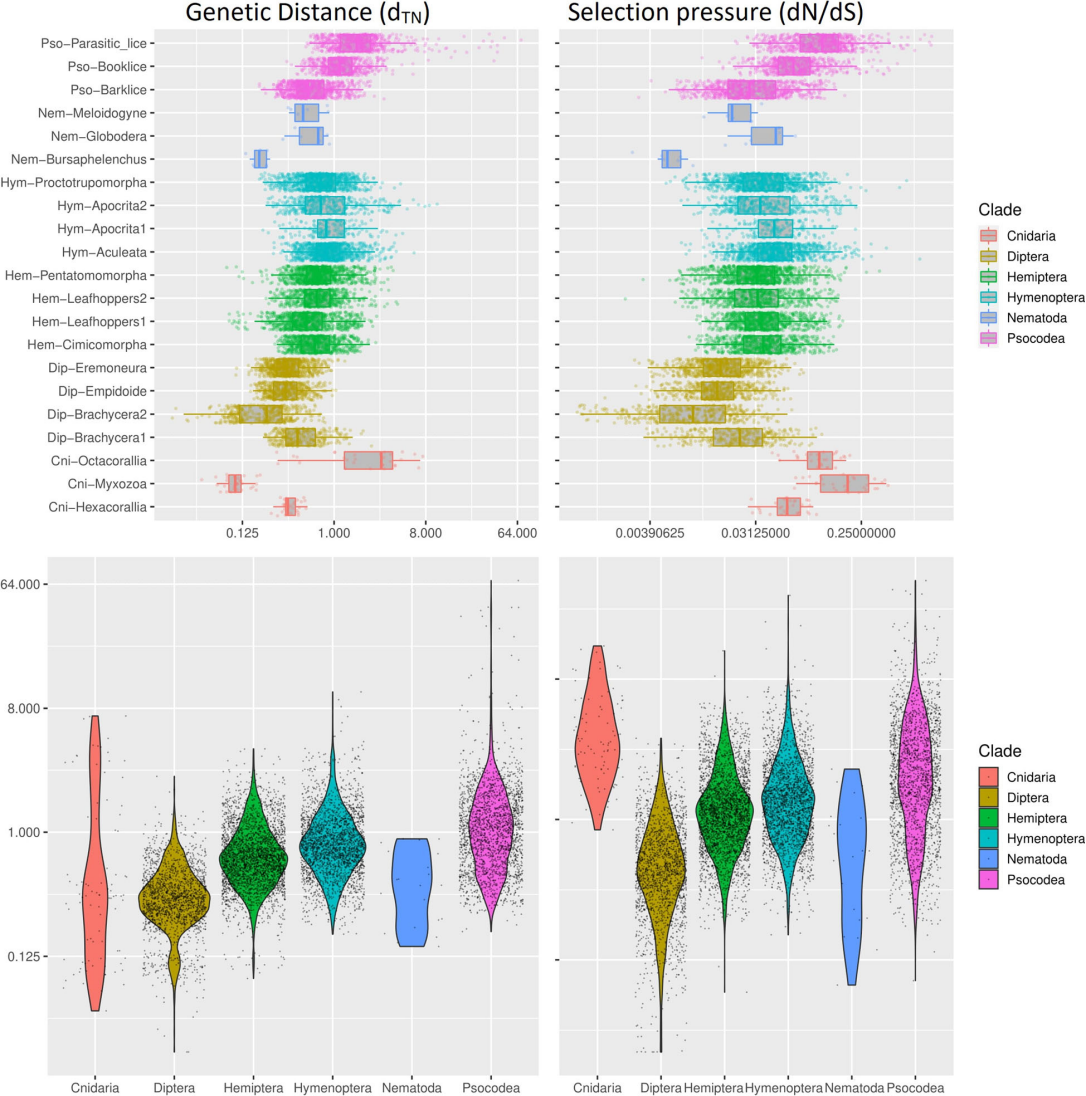
Comparison of d_TN_ and selection pressures across similar clades. The figure shows a comparison of genetic distance and selection pressure among multiple similar clades. Each datapoint in the figures is computed from pairwise comparisons of each protein-coding mt-gene across all the species in a clade. Although the datapoints are the same, the boxplots highlight the differences between clades, while the violinplots highlight the difference across clades and phyla. The numeric axis in each figure is measured as either Tamura-Nei Distance or dN/dS, and the axes have the same values in both boxplots and violinplots. The statistical tests performed on each clade can be seen in Additional file 3: Table S4

To investigate the cause of enhanced mt-genome structural variation, we performed a comparative analysis using the mt-genomes of the 15 insect clades mentioned before and species from Nematoda and Cnidaria. As mt-genome fragmentation is a rare event, we included examples from the phyla Nematoda and Cnidaria to have more species with fragmented mt-genomes and be able to better understand mt-genome fragmentation across the broader metazoan phylogeny. In the phylum Nematoda, we analysed the genus *Globodera*, in which at least three fragmented mt-genomes have been reported [16, 26, 27], along with its closely related species (Additional file 2: Table S1). In the phylum Cnidaria [11], we analysed two orders containing species with normal circular mt-genomes, and one order, Myxozoa, which contains species with fragmented circular mt-genomes (Additional file 2: Table S1). In this comparative analysis, we focused on two aspects, sequence divergence and the selection pressure on the mt-genes. We probed patterns across eleven protein-coding genes for the arthropods, seven for the nematodes and five for the Cnidaria because several genes were not present in some species of certain clades. For example, the mt-genomes (2 minichromosomes) of the myxozoan *Kudoa iwatai* possess only a few of the protein coding genes present in Arthropoda, while the rest of the genome is full of repeats and uncharacterized proteins. We used the Tamura-Nei distance (d_TN_) with the gamma correlation as a proxy to measure sequence divergence in mt-genes, and dN/dS as a proxy to measure history of selection. Through these proxies, we first performed a quantitative comparison between Psocodea (including barklice, booklice and parasitic lice) and three major insect clades (Diptera, Hymenoptera and Hemiptera) in which no mt-genome fragmentation has been detected (Fig. 4). To compare divergence and selection, we performed pairwise comparisons between the species in each clade and then compared the results across all clades. We believe this comparative method to be reliable, because it allows us to compare the differences within clades of the same age and similar species numbers. The result of the comparison is that almost all clades are significantly different from each other, with a few exceptions of clades within one order (e.g. Hymenoptera) that are not significantly different from each other (e.g. Aculeata and Aprocrita1). Similarly, when pooling the species together and dividing them into orders and phyla, the comparative analysis shows that all orders and phyla have significantly different selection pressure and genetic distance from each other. The results of all the statistical tests can be found in the Additional file 3: Table S4. Despite all clades experiencing significantly different genetic distances and selection pressures, there is a pattern unique to the Psocodea clade. Indeed, while all clades show a similar pattern between genetic distance and selection pressure, in the three Psocodea clades there is a clear increase in genetic distance as the purifying selection relaxes (i.e. tends to 1) that follows the level of mt-genome fragmentation. This pattern is only partially shared by the other two phyla (Nematoda and Cnidaria) harbouring species with fragmented mt-genomes. Indeed, while both clades with fragmented mt-genomes have a relaxed purifying selection, relative to the other species in the same group, their genetic distance does not increase in the same way, being similar or lower than species belonging to the same phyla. These results align with theoretical expectations, as the relaxed purifying selection is expected to increase the number of mutations, and thus, genetic distance. However, the relaxation of purifying selection in the Psocodea clades with fragmented mt-genomes suggests that fragmentation might somehow be related to selection pressure.

**Fig. 5:**
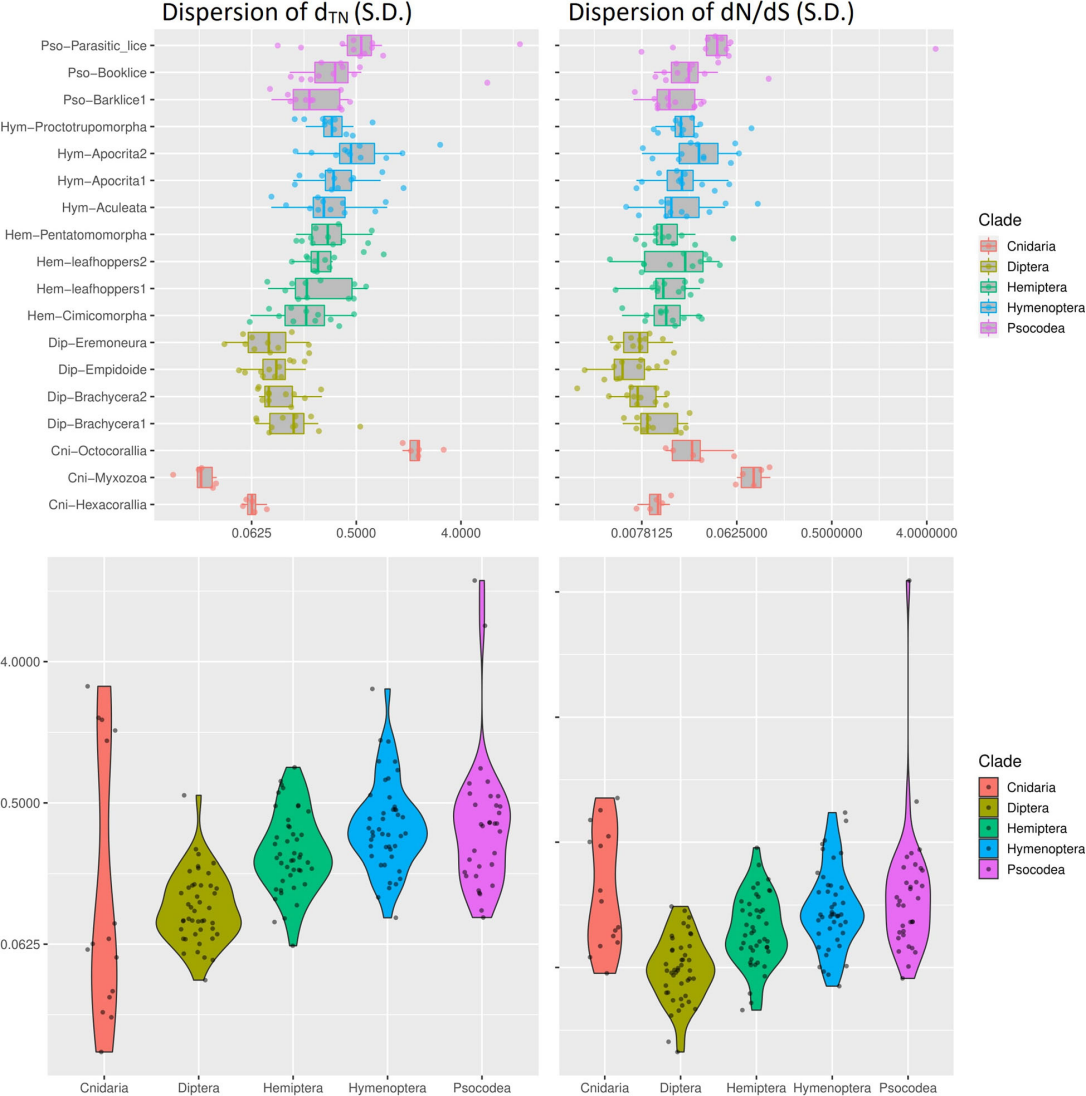
Comparison of d_TN_ and selection pressures variance across similar clades. The figures show a comparison of genetic distance and selection pressure standard deviation among multiple similar clades. We represented the standard deviation (S.D.) rather than the variance to make the figure easier to interpret, as squaring the S.D. would make the values hard to plots on the same axis. Each datapoint in the figures is computed from the S.D of each gene in the clade considered. For example, the four datapoints present in Cnidaria correspond to the S.D. of the four mt-gene analysed. Nematoda had only two genes conserved across all species, thus we excluded this phylum from the comparison. Although the datapoints are the same, the boxplots highlight the differences between clades, while the violinplot highlight the difference across clades and phyla. The numeric axis in each figure is the S.D. of either genetic distance or selection pressure, and the axes have the same values in both boxplots and violinplots. The statistical tests performed can be seen in Additional file 3: Table S4

To further investigate if the presence of species with fragmented mt-genomes within a clade are associated with the genetic distance and purifying selection within that clade, we investigated the variance of these measurements within each clade. However, as we had only two species for the three Nematoda clades, we could not include Nematoda in this analysis. The variance pairwise comparisons within and across clades showed that the variance of genetic distance and purifying selection pressure changes in a similar way within the most clades (Fig. 5, all the statistical tests can be found in the Additional file 3: Table S4). Indeed, only the species in the Cnidaria phylum and the parasitic lice had significantly different genetic distance and dN/dS variance from the other species in the same clade. Interestingly, although we saw in the previous analysis that the two measurements are significantly higher in the booklice (*Liposcelis*) when compared to the barklice (Fig. 4), the variance of these measurements is not significantly different between these two clades (Mann-Whitney *U* test, d_TN_ *p* value = 0.13, dN/dS *p* value = 0.3). This suggests that the “moderate” fragmentation (i.e. splitting the mt-genome in 2 or 3) *per se* does not significantly affect genetic distance and selection pressure. On the contrary, we can see that a higher level of fragmentation (i.e. splitting in many mt-genomes in parasitic lice) leads to much higher variability across both measurements. When comparing the variance of genetic distance and dN/dS across the different clades we saw that only some of them had the same variance, with only Diptera having significantly lower variance than all the other clades. The Psocodea order variance of genetic distance and dN/dS is not significantly different from Hymenoptera (Mann-Whitney *U* test, d_TN_ *p* value = 0.68, dN/dS *p* value = 0.48) but is significantly higher than all other clades. This is probably due to the presence of the parasitic lice in Psocodea, as the species in this clade all have relatively high divergence and relaxed purifying selection pressure. The lack of increased variance within the *Liposcelis* clade shows that the mt-genes across species in this clade are under similar forces (e.g. purifying selection) and probably had a similar evolution, despite some of the species within this clade having fragmented mt-genomes. Therefore, this analysis suggests a specific interpretation, where the mt-genome fragmentation is not the cause, but possibly a consequence, of the purifying selection relaxation and increased genetic distance. According to this interpretation, mt-genome fragmentation is a consequence of other forces that lower purifying selection and increase genetic distance. We propose our hypothesis on how this system works in the “Discussion” section.

## Discussion

This study provides the first compelling evidence of a link between sequence divergence, gene rearrangement and fragmentation. Our results show that across multiple metazoan clades, species that are more closely related to species with fragmented mt-genomes exhibit elevated sequence divergence and, while they are still under strong purifying selection, we see signatures of relaxed purifying selection associated with higher levels of mt-genome fragmentation. This phenomenon is most prominent in Psocodea, albeit present to a smaller degree in *Globodera* (Nematoda) and Myxozoa (Cnidaria). In Psocodea, the clade in which we have associated data on NGBs, the conservation of the mt-genome architecture decreases from barklice (no mt-genome fragmentation, few mt-gene rearrangements and moderate sequence divergence) to booklice (sporadic cases of fragmentation into 2 or 3 minichromosomes, frequent mt-gene rearrangements and high sequence divergence), culminating with parasitic lice (many cases of fragmentation with 8 to 20 minichromosomes, frequent mt-gene rearrangements and very high sequence divergence).

### The genus *Liposcelis*, a new model to study (mt) genome stability?

This study provides three lines of evidence that the non-model species in the genus *Liposcelis,* and its related clades in Psocodea*,* have unique features that make them good candidates for further studies on mt-genome stability. The first line of evidence is that the genus *Liposcelis* has a much higher level of mt-gene rearrangement when compared to any other clades analysed. This feature defies common knowledge [21, 28], as insects are usually species with slow evolving genomes [19], while the genus *Liposcelis* has some of the fastest evolving mt-genomes. The second line of evidence is that the d_TN_ and dN/dS measurements in the genus *Liposcelis* demonstrate that the mt-genome is quickly evolving despite being under strong purifying selection. Indeed, while the selection pressure relaxes compared to the barklice, it is still very close to 0 (96% of genes have dN/dS of less than 0.1) and only slightly higher than other Insecta clades. This phenomenon suggests that somehow other forces are driving the increased mutation rate in this species, providing a perfect system to uncover new insights into mt-genome evolution. The type of forces at play remains unknown; however, this effect might be explained by a faster mt-genome duplication rate, which would increase the number of mutations while keeping strong purifying selection. The third line of evidence for using this species as a model to study mt-genome stability is the presence of mt-gene recombination across populations of the same species, allowing analysis at multiple taxonomic scales. This unique feature makes it possible to track the recombination events and provide reliable evidence of *in vivo* mt-genome recombination. Nonetheless, the unique features of inter- and intraspecific mt-genome structural variation make this genus a good candidate in exploring genome structural variation. Given that the proteins involved in the DNA repair system, which regulate recombination processes, are shared across the nuclear and mitochondrial genomes, any mutations leading to an increase in recombination rate would be expected to affect both genomes [29, 30]. Thus, it is intriguing to investigate whether the recombination mechanism is limited to the mt-genome or it is a general phenomenon, present even within the nuclear genome. These features make the genus *Liposcelis* a, perhaps unique, system to investigate genome structural variation and (mt) genome stability.

### Conserved non-coding regions and diversified mt-gene rearrangement

This study revealed for the first time the presence of highly conserved regions within the mt-genome that are not coding for proteins involved in OXPHOS. Indeed, we provide conclusive evidence that populations of the species *L. bostrychophila* have highly conserved DNA motifs up to 100 nt long. Some of these motifs are longer than mt-protein coding genes such as ATP8, and more conserved [28]. In fact, several motifs have ~ 20% higher identity than mt-genes with similar length. While the reason behind the high sequence conservation within the non-coding region remains unknown, we highlight a few possible explanations. In our analysis, we uncovered the presence of conserved ORFs with unknown function, which is consistent with similar evidence from other studies that have reported novel proteins within the mt-genome [31–33]. Although we lack direct experimental evidence for the presence of these putative proteins, the conservation of these ORFs across both mt-chromosomes hints at a possible function. However, there are other possible explanations for the conservation of these sequences. One possible explanation for the presence of these conserved non-coding regions might involve the recombination of mt-genes. Indeed, the presence of identical regions next to mt-genes would facilitate their recombination across mt-chromosomes. We found that these conserved regions lie next to gene clusters that have been swapped across groups, supporting the hypothesis that these conserved regions are involved in facilitating homologous recombination. Despite the premise of mt-genome recombination remaining controversial, evidence from several metazoans suggests it may be pervasive [5, 15, 34, 35]. Indeed, we note that the position of mt-tRNAs is not well conserved across closely related species [34–36], suggesting the presence of a recombination mechanism for small genes in the mt-genome. If this is the case, then the booklice might have an enhanced version of this mechanism, and below we formulate hypotheses based on this possibility.

### Hypothesis: Recombination plays an important role in mt-genome fragmentation across animal clades

Based on the information gathered on the conserved DNA motifs, we suggest that their function is to regulate recombination in *Liposcelis*. Indeed, as mt-tRNA positions change across closely related Metazoa [36, 37], it is possible that a similar recombination mechanism that shuffles the position of small genes throughout the mt-genome may operate pervasively across metazoans. Accordingly, we hypothesise that this mechanism may contribute to the evolution of mt-genome fragmentation generally across the metazoan phylogeny, wherein errors during recombination might lead to unequal sorting of genes, and on occasion to the creation of new and fragmented mt-minichromosomes. But, what general features of mitochondrial genome architecture would be required to precipitate cases of mt-genome fragmentation, while still maintaining mt-genome functionality? Our study tried to find an answer by comparing whether clades that host members with mt-genome fragmentation exhibit consistent differences in genome architecture—be it substitution rate or selection pressure—relative to clades that do not host members with fragmentation, which may precipitate incidences of low genome stability within these clades. The results of the study led us to two insights.

The first insight is that episodes of mt-genome instability and fragmentation may be driven by accelerated evolution of the mtDNA genome, fuelled by a higher mutation rate. Indeed, Sloan et al. (2012) previously reported that species within the seed plant genus *Silene* with fragmented mt-genomes have mt-genes exhibiting heightened sequence divergence from their neighbouring species, suggesting that mutation rate might either influence, or be influenced by, mt-genome fragmentation [38]. Accordingly, we observed that the mt-genomes of metazoan species with fragmented mt-genomes, and their closest relatives, accumulate nucleotide substitutions at a faster pace than more distantly related species. This will culminate in a higher sequence divergence in mt-genes between species that are closely related to species with fragmented mt-genomes when compared to between species that are unrelated to any species exhibiting mt-genome fragmentation.

The second insight is based on the observation that despite a heightened substitution rate, the mt-genomes of species exhibiting high levels of mt-genome instability and sporadic fragmentation are nonetheless under strong selection (albeit somewhat relaxed relative to species with stable genomes), thus ensuring that any newly fragmented minichromosomes that spread through a population remain functional. Such selection will likely balance episodes of selfish selection and purifying selection. Within a scenario of heteroplasmy (multiple mtDNA molecules competing within an individual), fragmented minichromosomes will be expected to replicate more quickly and outcompete their single mt-chromosome counterparts within the germline of an individual [39–41]. Such selection would presumably be selfish in origin, fuelled by the replication advantage of the smaller minichromosomes [42]. Notwithstanding, purifying selection at the level of the organism and population would be intense to ensure that these minichromosomes remained functional and prevent degradation of respiratory performance [43]. However, this process could somehow be affected by the number of minichromosomes present. In fact, we observed a gradual relaxation of purifying selection pressure in Psocodea, along with an increase of minichromosomes, suggesting that splitting the genes involved in respiratory function across multiple minichromosomes can affect the selection pressure on these genes.

## Conclusions

In this work, we established a link between sequence divergence, recombination and fragmentation in the mitochondrial genomes of metazoans, the associations of which support a new hypothesis for the origin of the mt-genome fragmentation. Specifically, our results indicate that decreases in gene order stability in the mtDNA across the metazoan phylogeny are driven by heightened mutation rates in these clades, and that this sporadically leads to mt-genome fragmentation in some members of these clades. Moreover, we uncovered the presence of conserved non-coding regions in the mt-genomes of booklice, which are likely to regulate the numerous changes in mt-gene order between and even within species in this genus, through a recombination mechanism that we propose is likely to be general across metazoans.

## Methods

### Mt-genome data collection

All sequences used in phylogeny, gene boundary, sequence divergence and selection pressure analyses are listed in Additional file 2: Table S1. All mt-genomes, excluding newly sequenced *Liposcelis* data, were downloaded from the NCBI database. We first compared the number of shared gene boundaries (NGBs) across bilaterians, where genera with high quality sequenced genomes were chosen as representative taxa for their clades. That is genus *Drosophila* for insects, *Caenorhabditis* for nematodes, *Taenia* for cestodes, *Mytilus* for clams, *Oreochromis* for fish and *Mus* for mammals. We then selected clades with similar age to booklice (~ 200 MYA) from three close insect orders (Hymenoptera, Hemiptera, Diptera) for NGBs calculation and comparison. Furthermore, mt-genomes from these insects were leveraged to verify if the unusual high d_TN_ and strong purifying selection of booklice is an exception relative to other insects. We selected no more than two sequences from one genus to avoid overrepresentation and about ten sequences were ultimately picked in each of the four insect orders. Finally, several mt-genomes of Cnidaria and Nematoda were collected to enable us to investigate and disentangle possible connections between sequence divergence, mt-gene rearrangement and mt-genome fragmentation across diverse metazoan taxa.

### Booklice mt-genome sequencing, assembly and verification

We sequenced 9 mt-genomes from six species of booklice, five of which (*L. fusciceps*, *L. obscura*, *L. pearmani*, *L. rufa* and *L. corrodens*) were newly sequenced species, with four strains of *L. bostrychophila* re-sequenced (Additional file 2: Table S1). All booklice samples, before DNA extraction, were stored in 100% ethanol at − 80 °C. Genomic DNA was extracted using 20 individuals from each species (strains) using DNeasy Blood and Tissue Kit (QIAGEN). All genomic DNA was sent to the BerryGenomics company (Beijing, China) for library construction and sequencing. Two booklice, *L. fusciceps* and *L. obscura*, were sequenced on an Illumina Hiseq 2500 sequencer with the insert size of 450 bp and 250 bp paired-end sequencing. All others were sequenced on an Illumina Hiseq X10 sequencer with the insert size of 250 bp and 150 bp paired-end sequencing. All sequencing data were deposited in NCBI SRA with the ID PRJNA787771.

To generate five new mt-genome sequences, we applied two methods. The first method was to assemble genome drafts using IDBA-UD (PE250: 98% similarity, mink = 140, maxk = 240, step = 20; PE150: 98% similarity, mink = 80, maxk = 140, step = 20) [44]. The universal primer LCO1490-HCO2198 [45] was used to amplify partial *cox1* sequences of the five newly sequenced booklice. After sequencing, all *cox1* sequences were used to track the mt-genome like contigs from the genome drafts. Simultaneously, we applied a second method that mapped all sequencing reads to the partial *cox1* sequences for 1000 iterations using “Map to Reference” function in Geneious 10.1.3 [46]. Both methods lead to the same mt-genome sequence. Finally, to verify the authenticity of assembled mt-genomes, three booklice were randomly selected (*L. rufa*, *L. pearmani* and *L. corrodens*) for long PCR verification. For each species, three pairs of Long-PCR primers (Additional file 2: Table S5) were designed and their amplicons had overlapping regions on both sides, which together covered the whole mt-genome chromosome (agarose gel electrophoresis results in Additional file 1: Fig. S3). The full description of the method, reaction reagents and amplification programs for regular and long PCR followed Feng et al. [21].

The authors of a previous study [21] identified three *L. bostrychophila* groups with different mt genotypes. For the four newly sequenced *L. bostrychophila* strains, we did not know to which group each strain belonged. Thus, the four Illumina sequencing data were mapped to representative mt-genomes of each group (BJ strain of group 1, SY strain of group 2 and BB strain of group 3) using “Map To Reference” function in Geneious 10.1.3 [46]. Since the mt-genome sequence similarity among the three groups is about 80%, we set the parameter “Minimum Overlap Identity” during the mapping process to 90%. As a result, each of the new strains could map to only one representative mt-genome type. The four newly sequenced strains are distributed across all three groups: GR and ZZ strains belong to group 1, PR strain belongs to group 2 and AH strain belongs to group 3.

Annotations of all mt-genomes were conducted initially by the MITOS web server [47] and MitoZ [48]. Protein coding genes and ribosome RNA (rRNA) genes were then confirmed by BLAST searches of the NCBI database as well as alignment with homologous genes from other published booklice mt-genomes. ARWEN [49] and tRNAscan [50] were also used to confirm transfer RNA (tRNA) genes.

### Number of shared gene boundaries (NGBs) comparison

All gene boundaries were identified manually, with the recognition principle and an example outlined in Additional file 1: Fig. S1. In principle, we counted only the adjacent genes with the same relative transcription direction as one shared gene boundary for the two species (e.g. *atp6atp8* and *atp6atp8*; *atp6atp8* and *atp8atp6*). The number of shared gene boundaries was counted as the NGBs between them. All tRNA genes were excluded from NGBs analysis due to their pervasive mobility. Pairwise NGBs were extracted in each selected taxon. For a general view on NGBs distribution across Bilateria (shown in Fig. 1B), pairwise NGBs calculation was conducted in (1) six representative taxa, including six sequences in *Mus*, seven in *Oreochromis*, six in *Mytilus*, seven in *Taenia*, thirteen in *Caenorhabditis* and eleven in *Drosophila* and (2) our focal target, genus *Liposcelis* with 11 mt-genomes. Pairwise NGBs were then calculated on four orders of insects (shown in Fig. 1C) with an age of ~ 200 MYA (details will be introduced in *Sequence divergence and selection analyses* part), including Hemiptera (50 sequences), Hymenoptera (43 sequences), Diptera (40 sequences) and Psocodea (31 sequences). Based on canonical Insecta phylogeny [51], these orders are phylogenetically close to Psocodea with many sequenced mt-genomes. In Fig. 2A, we calculated the NGBs of booklice with or without mt-genome fragmentation: all eleven mt-genome sequences in *Liposcelis* were incorporated in *Liposcelis*-All; four fragmented mt-genomes (*L. bostrychophila* BJ strain, *L. paeta*, *L. entomophila*, *L. brunnea*), were used for *Liposcelis*-Fragmented; and the other seven were used in *Liposcelis*-Intact. For comparison of NGBs between different clades, Mann-Whitney *U* test was conducted using R 3.5.0.

### Multiple sequence alignment and phylogenetic analyses

We inferred the phylogenetic relationship of *Liposcelis* species using mt-genome sequences (species marked in Additional file 2: Table S1, phylogenetic tree in Fig. 2D, Additional file 1: Fig. S2). For *L. bostrychophila*, BJ (Beijing, China), HLM (Huangliangmeng, Hebei, China) and BB (Beibei, Chongqing, China), were selected as representative sequences of intra-specific group 1, group 2 and group 3, respectively. We used the barklouse, *Stimulopalpus japonicus*, as the outgroup of the analysis because it is the closest species to genus *Liposcelis* with a known mt-genome. Only protein-coding genes as well as rRNA genes were considered and concatenated into four datasets: (1) dataset “PCG123rRNA”, in which there are concatenated 12 protein-coding genes (*atp6*, *atp8*, *cox1*, *cox2*, *cox3*, *cob*, *nad1*, *nad2*, *nad3*, *nad4*, *nad5*, *nad6*) and two rRNA genes (*rrnL* and *rrnS*); (2) dataset “PCG123”, including only 12 protein-coding genes; (3) dataset "PCG12rRNA”, in which only the first and second codon positions of the 12 protein-coding genes and two rRNA genes are included; and (4) dataset “PCG12”, in which there are concatenated the first and second codon positions of the 12 protein-coding genes. Due to the absence of *nad4L* in *L. entomophila* and *L. sculptilimacula*, it was excluded from phylogenetic analysis. Each protein coding gene was aligned individually on codon mode using the MAFFT method [52] with the L-INS-I algorithm, which was implemented in TranslatorX [53]. MAFFT was also used to align two rRNA genes leveraging the G-INS-I strategy. All ambiguous sites were removed with Gblocks v0.91b [54]. Sequences were then concatenated using the “concat” function of the SeqKit toolkit [55]. Abovementioned sequence processing was conducted in a local Linux server.

We performed a phylogenetic analysis on the four concatenated datasets using Bayesian (BI) and maximum likelihood (ML) methods, leveraging MrBayes 3.2.6 [56], IQTREE [57] and PhyML 3.0 [58]. For the BI analysis, PartitionFinder was used to select the best nucleotide substitution models. The settings on MrBayes included two independent sets of Markov chains (one cold and three heated chains), 10 million generations, sampling every 1000 generations, and 25% burn-in. The ML analysis was performed using two different tools. IQ-TREE web server was used while optional evolutionary models were detected with the “Auto” option by which the best substitution models were detected automatically. The number of ultrafast bootstraps was set to 1000. In PhyML, the best evolutionary models were selected using the “Smart Model Selection” method based on Bayesian information criterion. PhyML phylogenetic trees were constructed with a bootstrap of 1000 replicates. Optimal nucleotide substitution models for all BI and ML analyses are listed in Additional file 2: Table S6.

### Motif related analyses

The motif conservation was calculated using the MEME suite online [59] with the following command line ‘-dna -oc . -nostatus -time 18000 -mod zoops -nmotifs 6 -minw 50 -maxw 300 -objfun classic -minsites 40 -revcomp -markov_order’. The conserved regions were extracted from the output and plotted using WebLogo3 (http://weblogo.threeplusone.com/create.cgi).

### Sequence divergence and selection analyses

All sequences used in the analyses of d_TN_ and dN/dS are described in Additional file 2: Table S1. Multiple sequence alignments were conducted on each dataset following the procedure outlined in *Multiple sequence alignment and phylogenetic analyses*. Two genes (*atp8* and *nad6*) were discarded in the following analyses because after the multiple sequence alignment, their sequence lengths are shorter than 30 bp and as such mutations of one nucleotide would have a disproportionate effect on the alignment. For the other 11 genes (*atp6*, *cob*, *cox1*, *cox2*, *cox3*, *nad1*, nad2, *nad3*, *nad4*, nad4l, *nad5*), we performed d_TN_ extraction as well as dN/dS calculation using MEGA [60] and KaKs_Calculator [61, 62]. The d_TN_ was calculated using MEGA 7 using the Tajima-Nei model with gamma correlations. The default model averaging (MA) method was used in KaKs_Calculator for each protein coding gene in different datasets. The data generated were used for the statistical tests computed through the rank-sum function on MATLAB. This function performs a test equivalent to the Mann-Whitney *U* test, and the results of the analyses can be all found in Additional file 3: Table S4.

The first dataset includes four Insecta orders (Hemiptera, Hymenoptera, Diptera, Psocodea). To better understand variations in order level, those clades with an age of ~ 200 MYA were selected based on published phylogeny. All the clades, species, clade ages and sequences GenBank accession numbers were listed in Additional file 2: Table S1. Specifically, the overall phylogenetic relationship among the four orders was based on Misof et al. [51]. Phylogenetic relationship of Diptera was based on Wiegmann et al. [63] where the selected clades were (Brachycera1 + Brachycera2) + (Empidoidea + Eremoneura1). The updated phylogeny of Hymenoptera is by Peters et al. [64]. The phylogeny of selected Hymenoptera clades could be well reflected by (Aculeata + Apocrita1) + (Proctotrupomorpha + Apocrita2). For Hemiptera and Psocodea, a recent systematic phylogenetic analysis was conducted by Johnson et al. [65] The clades selected could be well characterised by (Pentatomomorpha+Cimicomorpha)+(leafhoppers1+leafhoppers2) for Hemiptera and Barklice1 + (Booklice + Parasitic lice) for Psocodea.

The other dataset includes two clades (Cnidaria and Nematoda) with fragmented mt-genomes. In Cnidaria, the phylogeny was based on Yahalomi et al. [11]. We excluded those special linear mt genomes which might exhibit a different evolutionary path compared to circular ones. Thus, the three clades (Myxozoa + (Hexacorallia + Octocorallia)) are selected. In Nematoda, the presence of fragmented mt-genomes is limited to the order Tylenchid whose phylogeny is already reconstructed [16, 66]. We tried to balance the number of sequences across all taxa, however, the genus *Gblobodera* has only two available mt-genomes. Thus, we selected the two sequences from close taxa *Meloidogyne* (root knot nematode) and *Bursaphelenchus* as well, forming (*Bursaphelenchus* + (*Gblobodera* + *Meloidogyne*)).

## Supplementary Information

**Additional file 1: **Fig. S1. The sketch map for NGBs counting. (A) A schematic representation of the rationale behind the calculation of NGBs. We counted only the adjacent genes with the same relative transcription direction as one gene boundary. (B) An example of NGBs counting. The circular chromosome A and B have five genes (gene A, gene B, gene C, gene D, gene E). The gene boundaries for species A are A-B, B-C, C-D, D-E, E-A while those for species B are A-B, B-C, C-E, E-D, D-A. The only matched gene boundary between the two species is A-B. So the NGBs between A and B is 1. We used the same method to calculate the NGBs between two mitochondrial genomes. Fig. S2. Newly constructed phylogenies of booklice. We inferred the phylogenetic relationship of *Liposcelis species* using mt-genome sequences with four datasets and three softwares. Details were introduced in “Materials and Methods” section. The numbers in each node represents BI posterior probability or ML boostrap value for each datasets/methods: PCG123rRNA/MrBayes+PCG123/MrBayes+PCG12rRNA/MrBayes+PCG12/MrBayes+PCG123rRNA/IQtree+PCG123/IQtree+PCG12rRNA/IQtree+PCG12/IQtree+PCG123rRNA/PhyML+PCG123/PhyML+PCG12rRNA/PhyML+PCG12/PhyML. The best substitution models for different methods are listed in Additional file 3: Table S4. All three methods supported the same topology among booklice species. Fig. S3. Agarose gel electrophoresis Long-PCR products of the three booklice. (A) Agarose gel electrophoresis PCR products of *L. corrodens* KS strain. Amplicons 1-6 are from LcCZLF/LcCZLR, LcCZC1F/LcCZLF, LcCZC1R/LcCZLR, LcCZLF/LcCZLR, LcCZC1F/LcCZLF and LcCZC1R/LcCZLR. Marker (M): 1 kb DNA ladder marker: 10 kb, 8 kb, 6 kb, 5 kb, 4 kb, 3 kb, 2.5 kb, 2 kb, 1.5 kb, 1 kb, 700 bp, 500 bp, 300 bp. 1 μL Long-PCR product was used for every sample of each run. (B) Agarose gel electrophoreses PCR products of *L. pearmani* KS strain. Amplicons 1-3, 5-8 are from LpearC1F/LpearC1R, LpearLF/LpearLR, LpearSF/LpearSR, LpearLF/LpearSF, LpearLR/LpearSR, LpearC1F/LpearSR and LpearC1R/LpearSF. (C) Agarose gel electrophoreses PCR products of *L. rufa* KS strain. Amplicons 1-5 are from LruLF/LruLR, LruLF/LruSR, LruLR/LruSF, LruC1F/LruSF and LruC1R/LruSR.

**Additional file 2: **Table S1. Animal mitochondrial genomes used in this study. Table S2. Blast results from six motifs from the non-coding region of *Liposcelis bostrychophila*. Table S3. Blast results of open reading frames recognized from six motifs. Table S5. Long-PCR primers used to verify three booklice mt genomes. Table S6. Optimal models in phylogenetic analyses.

**Additional file 3:.** Table S4. Statistics in sequence analyses.

## Data Availability

All data generated or analysed during this study are included in this published article, its supplementary information files and publicly available repositories. Sequencing data for Booklice mt-genome was deposited in NCBI SRA with the ID PRJNA787771.
