# Supplementary material for: Fragmentation in mitochondrial genomes in relation to elevated sequence divergence and extreme rearrangements
Source: BMC Biol. 2022 Jan 7;20:7. doi: 10.1186/s12915-021-01218-7 (PMC8742463; doi:10.1186/s12915-021-01218-7)
Supplement: Supplementary file 1 — Additional file 1: Fig. S1. The sketch map for NGBs counting. (A) A schematic representation of the rationale behind the calculation of NGBs. We counted only the adjacent genes with the same relative transcription direction as one gene boundary. (B) An example of NGBs counting. The circular chromosome A and B have five genes (gene A, gene B, gene C, gene D, gene E). The gene boundaries for species A are A-B, B-C, C-D, D-E, E-A while those for species B are A-B, B-C, C-E, E-D, D-A. The only matched gene boundary between the two species is A-B. So the NGBs between A and B is 1. We used the same method to calculate the NGBs between two mitochondrial genomes. Fig. S2. Newly constructed phylogenies of booklice. We inferred the phylogenetic relationship of Liposcelis species using mt-genome sequences with four datasets and three softwares. Details were introduced in “Materials and Methods” section. The numbers in each node represents BI posterior probability or ML boostrap value for each datasets/methods: PCG123rRNA/MrBayes+PCG123/MrBayes+PCG12rRNA/MrBayes+PCG12/MrBayes+PCG123rRNA/IQtree+PCG123/IQtree+PCG12rRNA/IQtree+PCG12/IQtree+PCG123rRNA/PhyML+PCG123/PhyML+PCG12rRNA/PhyML+PCG12/PhyML. The best substitution models for different methods are listed in Additional file 3: Table S4. All three methods supported the same topology among booklice species. Fig. S3. Agarose gel electrophoresis Long-PCR products of the three booklice. (A) Agarose gel electrophoresis PCR products of L. corrodens KS strain. Amplicons 1-6 are from LcCZLF/LcCZLR, LcCZC1F/LcCZLF, LcCZC1R/LcCZLR, LcCZLF/LcCZLR, LcCZC1F/LcCZLF and LcCZC1R/LcCZLR. Marker (M): 1 kb DNA ladder marker: 10 kb, 8 kb, 6 kb, 5 kb, 4 kb, 3 kb, 2.5 kb, 2 kb, 1.5 kb, 1 kb, 700 bp, 500 bp, 300 bp. 1 μL Long-PCR product was used for every sample of each run. (B) Agarose gel electrophoreses PCR products of L. pearmani KS strain. Amplicons 1-3, 5-8 are from LpearC1F/LpearC1R, LpearLF/LpearLR, LpearSF/LpearSR, LpearLF/LpearSF, LpearLR/L [file 12915_2021_1218_MOESM1_ESM.pdf]

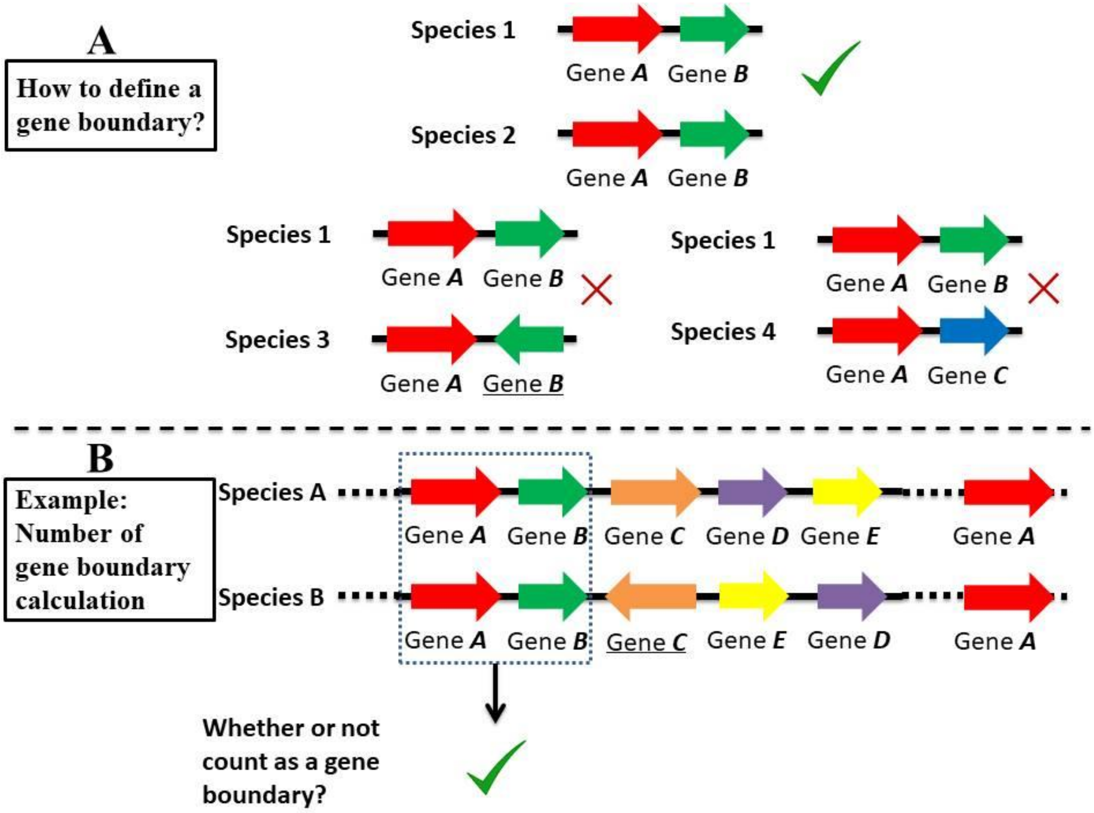

Fig. S1 The sketch map for NGBs counting. (A) A schematic representation of the rationale behind the calculation of NGBs. We counted only the adjacent genes with the same relative transcription direction as one gene boundary. **(B)** An example of NGBs counting. The circular chromosome A and B have five genes (gene A, gene B, gene C, gene D, gene E). The gene boundaries for species A are A-B, B-C, C-D, D-E, E-A while those for species B are A-B, B-C, C-E, E-D, D-A. The only matched gene boundary between the two species is A-B. So the NGBs between A and B is 1. We used the same method to calculate the NGBs between two mitochondrial genomes.

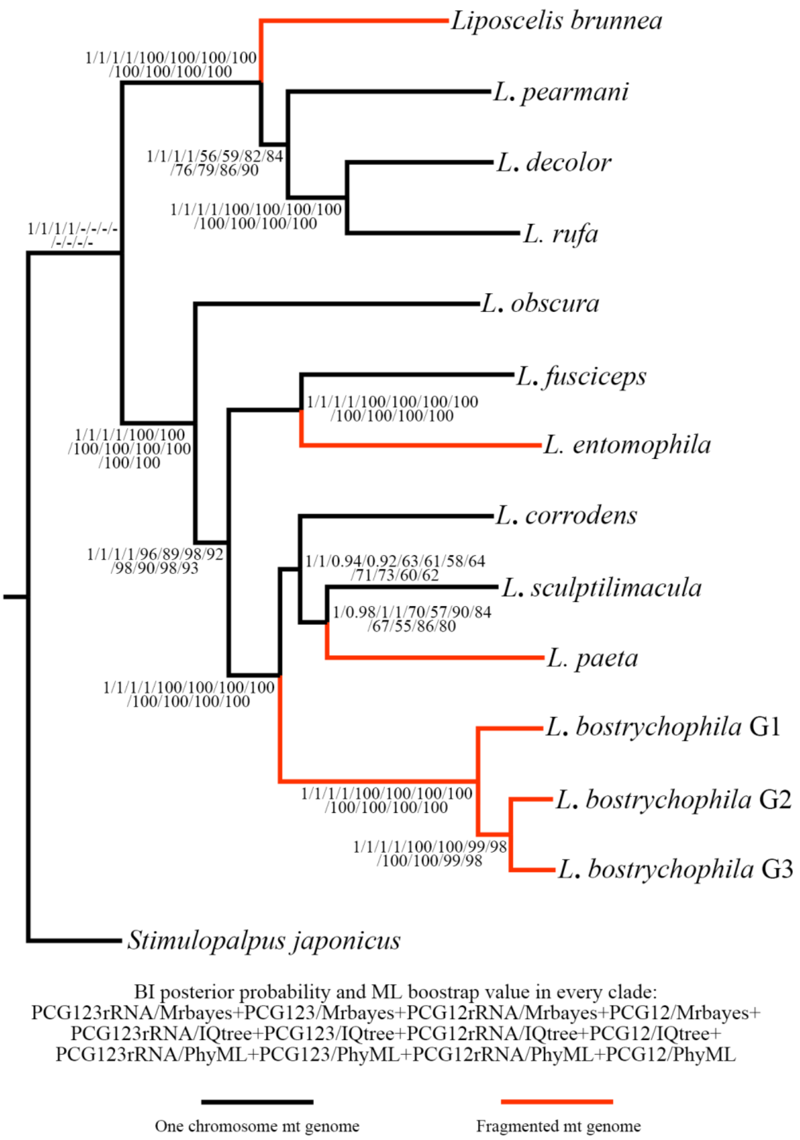

Fig. S2 Newly constructed phylogenies of booklice. We inferred the phylogenetic relationship of *Liposcelis species* using mt-genome sequences with four datasets and three softwares. Details were introduced in “Materials and Methods” section. The numbers in each node represents BI posterior probability or ML boostrap value for each datasets/methods:
PCG123rRNA/MrBayes+PCG123/MrBayes+PCG12rRNA/MrBayes+PCG12/MrBayes+PCG123rRNA/IQtree+PCG123/IQtree+PCG12rRNA/IQtree+PCG12/IQtree+PCG123rRNA/PhyML+PCG123/PhyML+PCG12rRNA/PhyML+PCG12/PhyML. The best substitution models for different methods are listed in Table S4. All three methods supported the same topology among booklice species.

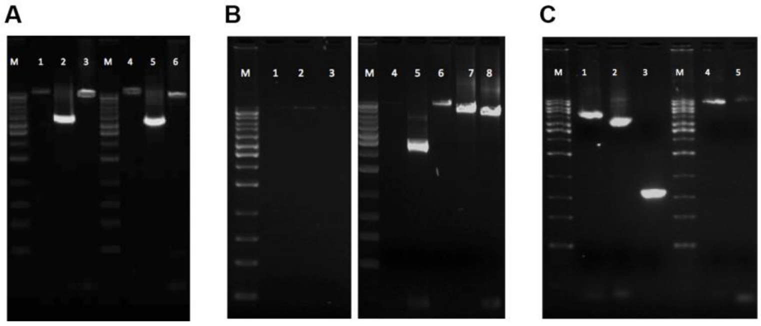

Fig. S3 Agarose gel electrophoresis Long-PCR products of the three booklice. (A) Agarose gel electrophoresis PCR products of *L. corrodens* KS strain. Amplicons 1-6 are from LcCZLF/LcCZLR, LcCZC1F/LcCZLF, LcCZC1R/LcCZLR, LcCZLF/LcCZLR, LcCZC1F/LcCZLF and LcCZC1R/LcCZLR. Marker (M): 1kb DNA ladder marker: 10 kb, 8 kb, 6 kb, 5 kb, 4 kb, 3 kb, 2.5 kb, 2 kb, 1.5 kb, 1 kb, 700 bp, 500 bp, 300 bp. 1 μL Long-PCR product was used for every sample of each run. **(B)** Agarose gel electrophoreses PCR products of *L. pearmani* KS strain. Amplicons 1-3, 5-8 are from LpearC1F/LpearC1R, LpearLF/LpearLR, LpearSF/LpearSR, LpearLF/LpearSF, LpearLR/LpearSR, LpearC1F/LpearSR and LpearC1R/LpearSF. **(C)** Agarose gel electrophoreses PCR products of *L. rufa* KS strain. Amplicons 1-5 are from LruLF/LruLR, LruLF/LruSR, LruLR/LruSF, LruC1F/LruSF and LruC1R/LruSR.
